# Chinese herbal medicine for the treatment of recurrent miscarriage: a systematic review of randomized clinical trials

**DOI:** 10.1186/1472-6882-13-320

**Published:** 2013-11-18

**Authors:** Guo-Yan Yang, Hui Luo, Xing Liao, Jian-Ping Liu

**Affiliations:** 1 Center for Evidence-based Chinese Medicine, Beijing University of Chinese Medicine, Beijing, China; 2 Institute for Tibetan Medicine, China Tibetology Research Center, Beijing, China; 3 Institute of Basic Research in Clinical Medicine, China Academy of Chinese Medical Sciences, Beijing, China

**Keywords:** Chinese herbal medicine, Recurrent miscarriage, Systematic review, Randomized clinical trials

## Abstract

**Background:**

Traditional Chinese medicine has been widely used for the treatment of recurrent miscarriage in China and other Asian countries for long time. We conducted this review to systematically summarize the evidences of Chinese herbal medicine (CHM) for the prevention and treatment of recurrent miscarriage in randomized trials, and evaluate the effectiveness and safety of CHM compared with placebo or conventional medicine.

**Methods:**

We searched studies in PubMed, ClinicalTrials, the Cochrane Library, CNKI, SinoMed and VIP databases until December, 2012. Randomized trials on CHM alone or in combination with conventional medicine for recurrent miscarriage compared with placebo or conventional medicine were included. We evaluated the methodological quality of each included trials using the Cochrane risk of bias tool.

**Results:**

A total of 41 RCTs (3660 participants) were included. The majority of trials had a high or unclear risk of bias. CHM used alone or plus progesterone-based treatment showed superior effect over progesterone-based treatment in improving live birth rate and embryonic developmental state (measured by B ultrasound). However, there is substantial heterogeneity within each subgroup analysis (*I*^*2*^ ranging from 35% to 71%). CHM plus progesterone and hCG-based treatment was superior to progesterone and hCG-based treatment in improving the embryonic developmental state, but not live birth rate. No severe adverse events were reported in relation to CHM.

**Conclusions:**

Some Chinese herbal medicines or in combination with progesterone-based treatment demonstrated potentially beneficial effect in improving live birth rate and embryonic developmental state for women with recurrent miscarriage. However, due to the substantial heterogeneity among the herbal interventions and limitations of methodological quality of the included trials, it is not possible to recommend any specific CHMs for recurrent miscarriage. Further rigorous clinical trials are warranted to evaluate the efficacy and safety of CHM.

## Background

Pregnancy loss is a common clinical problem in reproduction, occurring in 15% ~ 40% of reproductive-aged women. Recurrent miscarriage, defined as the loss of three or more consecutive spontaneous abortions [1,2], affects 1% ~ 2% women of reproductive age [2]. Moreover, in clinical practice, many clinicians define recurrent miscarriage as two or more losses; this increases the prevalence rate to approximately 5% of all couples trying to conceive [3,4]. Risk factors for recurrent miscarriage varies widely, such as maternal age, number of previous miscarriages, antiphospholipid syndrome, genetic factors, anatomical deformity of reproductive organs, endocrine disorders, immune factors, and infective agents, but in more than half of such patients, no certain diagnosis could be identified [4,5]. Recurrent pregnancy losses could bring physical and psychological harms to the patients, as well as a heavy economic burden, and could even lead to family and social problems. Hence, researches on the prevention and treatment of recurrent miscarriage are of significantly clinical and social importance.

Since the knowledge of etiology and pathogenesis of recurrent miscarriage is largely unclear, various interventions have been used in clinical practice, but the majority of them are still lack of sufficient evidence to support their use to prevent a miscarriage in women with recurrent miscarriage. Evidence from the guideline for the management of recurrent pregnancy loss published by American Congress of Obstetricians and Gynecologists (ACOG) [1] and Royal College of Obstetricians and Gynecologists (RCOG) [6] suggest that, except for the recommendation of low-dose aspirin plus with low-molecular-weight heparin for recurrent miscarriage patients with antiphospholipid syndrome, there is few good evidence to support the other commonly used interventions for recurrent miscarriage; some immunotherapies such as paternal cell immunisation, third-party donor leucocytes, trophoblast membranes and intravenous immunoglobulin in women with previous unexplained recurrent miscarriage do not improve the live birth rate, and have potentially serious adverse effects. Observation is a reasonable strategy for patients, and a subsequent pregnancy will result in a live birth for about two thirds of couples [1,6,7].

As no curative conventional interventions are available for the disease, many parents prefer to seek alternative medicine. In China and other Asian countries, traditional Chinese medicine (TCM) has been widely used for the treatment of recurrent miscarriage for a long time. With the dissemination and application of clinical epidemiology and evidence-based medicine in TCM during the past two decades, a series of trials evaluating the efficacy and safety of Chinese herbal medicine (CHM) for recurrent miscarriage were conducted, but the findings have not yet been systematically summarized. The objective of this review is to critically appraise the existing randomized clinical trials (RCTs) on CHM for the prevention and treatment of recurrent miscarriage, and provide evidence-based evaluation on the efficacy and safety of CHM for this condition.

## Methods

### Study search

A search strategy was designed to search all the available literature. We searched PubMed, ClinicalTrials, the Cochrane Library (Issue 10, 2012), the Chinese National Knowledge Infrastructure Databases (CNKI), the Chinese Science and Technology Periodical Database (VIP), the Chinese Biomedical Database web (SinoMed), and the Wanfang Database, from their inception to December, 2012. There was no limitation on language or publication type. The search terms included [“Abortion, habitual” and “Medicine, Chinese Traditional”] as Mesh terms and [“recurrent miscarriage” or “recurrent pregnancy loss” or “recurrent spontaneous abortion”] as “all fields” searching in PubMed, ClinicalTrials, and the Cochrane Library. In the Chinese databases, we employed recurrent miscarriage and randomiz* as the major search terms with no limitations on the modalities CHM employed.

### Study selection

Two authors (H. Luo and X. Liao) selected the literature independently. Papers were screened according to the title and then selected through abstracts. The full texts were retrieved if they potentially met the inclusion criteria.

Studies meeting the following criteria were included in this review: (1) type of study: randomized clinical trials (RCTs); (2) type of participants: females who had a history of at least two or more miscarriage, and were trying to get pregnant or were already pregnant; (3) type of interventions: the study was designed to compare the effectiveness and safety of CHM or CHM plus conventional medicine with placebo or conventional medicine; (4) type of outcomes: live birth rate (the primary outcome), embryonic developmental state (secondary outcome), and adverse events. The secondary outcome should be measured by type B ultrasound, presented as effective rate. “Effective” is defined as normal embryo development with the increasing weeks of gestation, and “ineffective” is defined as embryo stopping growing or recurrence of spontaneous abortion.

The following types of studies were excluded: (1) multiple publications reporting the same data of patients; (2) lack of basic information on participants or interventions; (3) controlled treatment included CHM therapies; as in this case, it would be impossible to evaluate the specific effects of the intervention.

If there was a lack of some important information in the paper, such as methodology, diagnosis, interventions and outcomes, we would try to contact the original authors to clarify the data.

### Risk of bias assessment

We conducted the selection of studies by using criteria from the Cochrane Handbook for Systematic Reviews of Interventions, version 5.0.2 [8]. Full texts were retrieved for any potentially relevant studies, and then were identified according to the inclusion criteria. Any disagreements were resolved by discussion or consulting to the third researcher.

The methodological quality of included RCTs was assessed using Cochrane risk of bias tool. The generation of the allocation sequence, allocation concealment, blinding and outcome reporting were taken into account for assessment. All the included trials would be categorized as low/unclear/high risk of bias: trials that met all the criteria were categorized as low risk of bias; those that met none of the criteria were categorized as high risk of bias; and the others were categorized as unclear risk of bias if insufficient information was available to make a judgment. Disagreements were consulted to the third author (JP Liu) to resolve.

### Data extraction

Two authors (G. Yang and H. Luo) extracted the data independently using a pre-designed data form. The following data were extracted: (1) citations (author, title, journal, year, issue, volume, and page); (2) methodological characteristics of trials; (3) participants (sample size, age, causes of recurrent miscarriage, and current status of patients); (4) detailed information of interventions and controls; (5) outcome measures; (6) a summary of results; and (7) adverse effects.

### Data analysis

The data were analyzed using RevMan 5.0 software. The effect measure was summarized by risk ratio (RR) with a 95% confidence interval (CI). Meta-analysis was used if the trials were homogeneous on study design, participants, interventions, control, and outcome measures. We used fixed effect model unless there are evidence of substantial heterogeneity where random effect model would be applied for pooling data. We assessed heterogeneity using both the I-squared statistic, and considered an I-squared value greater than 50% indicative of substantial heterogeneity [8]. If sufficient number of trials (i.e., over 10 trials), we would conduct funnel plot to detect potential publication bias.

## Results

**Figure 1:**
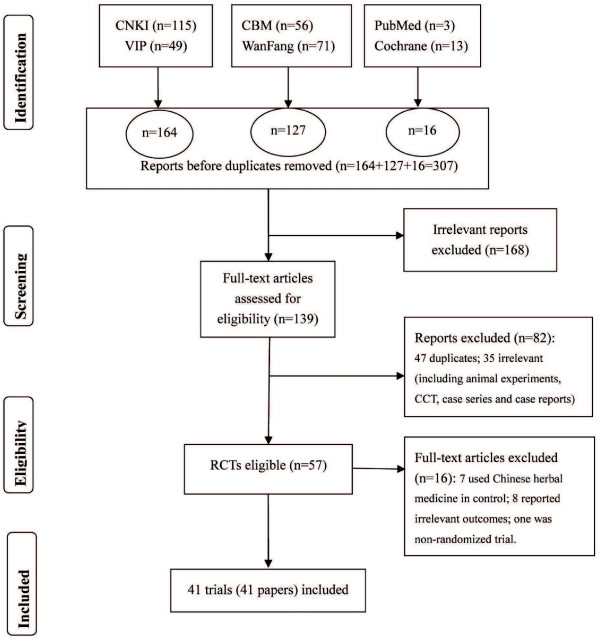
**Flowchart of study searching and selection.** Presentation of the process of study searching and selection.

Using the search strategy, we identified a total of 307 references. After screening titles and abstracts and excluded duplicated papers, 57 full papers were downloaded for eligibility identifying. Finally, 16 studies were excluded with reasons, and 41 RCTs were included in this systematic review (Figure 1).

### Description of included trials

All of the 41 trials were conducted in China, and published in Chinese journals. A total of 3660 women with recurrent miscarriage were involved, with an average number of 89.3 per trial, ranging from 31 to 185. Among the included trials, 41.5% (17/41) reported participants with the loss of two or more consecutive spontaneous abortions, and 56.1% (23/41) reported participants with three or more consecutive spontaneous abortions. One trial didn’t report the definition of “recurrent miscarriage”. 25 trials reported the cause of recurrent miscarriage, including antiphospholipid syndrome [9-12], negative blocking antibodies [13], luteal phase defect [14,15], hyperprolactinemia [16], pre-thrombosis [17], cytomegalovirus infection [18], and unexplained reasons [19-33]. Participants in 26 trials (63.4%, 26/41) were already pregnant; in 11 trials (26.8%, 11/41) were trying to be pregnant; females both already pregnant and trying to be pregnant were included in two trials (4.9%, 2/41) [34,35]; another two trials (4.9%, 2/41) [22,28] didn’t report this item.

CHM included patent medicine in three trials [16,36,37], and practitioner-prescribed herbal formula based on TCM syndrome differentiation in 38 trials, of which 7 trials [10,13,17,29,32,38,39] prepared the CHMs in hospital preparation room. The preparation of CHM included decoction (82.9%, 34/41), pill (7.3%, 3/41), granule (4.9%, 2/41), powder (4.9%, 2/41), capsule (2.4%, 1/41), oral liquid (2.4%, 1/41) and ointment (2.4%, 1/41). The most frequently used decoction was modified ‘*Shou Tai Wan*’ (21.9%, 9/41), and other formulas were prescribed according to the experience of physicians and clinical presentations/syndromes of the patients. Five trials [9,12,17,37,40] applied CHMs with the action of invigorating blood, and the most frequently used compositions in these CHMs were Radix Angelicae Sinensis (*dang gui*) [9,12,17,37,40] and Radix et Rhizoma Salviae Miltiorrhizae (*dan shen*) [9,17,40]. The detailed compositions of all CHMs for recurrent miscarriage were presented as supporting information (see Additional file 1: Table S1).

Conventional medicines included progesterone, human chorionic gonadotrophin (hCG), heparin sodium, folic acid, vitamin C, vitamin E, aspirin, prednisone, dydrogesterone, immunotherapy, and other symptomatic supports. For treatment durations, 27 trials treated patients from trials start to the 3^rd^ to 4^th^ month of gestation, or one week or half a month exceeding the previous miscarriage time, 7 trials lasted for 10 days to 3 months, and one trial [41] treated patients till birth, while another 6 trials were unclear. 21 trials (51.2%, 21/41) reported live birth rate and the remaining 20 trials (48.8%, 20/41) reported the status of embryonic development. More details of the trials were presented in Additional file 2: Table S2.

### Methodological quality

A majority of trials was of high or unclear risk of bias, indicating poor methodological quality. The randomized allocation of participants was mentioned in all trials; however, only 6 trials reported the methods of sequence generation, including using random number table [14,20,21,37,40] and card drawing [16], of which none reported allocation concealment. One trial used matched placebos to blind participants and practitioners [36], in which patients in the intervention group received both real CHM oral liquid and placebo of progesterone, while participants in the control group received both real progesterone and placebo of CHM oral liquid. The number of dropouts and intention-to-treat analysis were not reported in all trials. Meanwhile, in 11 trials which recruited the patients trying to get pregnancy, only two trials reported pregnancy rate [16,18]. None of the trials reported sample size estimation. The baseline information in nine trials not adequately reported the age or times of previous miscarriage [9,12,26,28,36,38,42-44]. Since the protocols of all trials were not available, we assessed the selective reporting bias by comparing their outcome measures in methods and the reporting of results, and they had a low risk of bias (Additional file 3: Figure S1 and Additional file 4: Figure S2).

To clarify the data, we tried to contact the original authors of those trials. Since few included papers provided corresponding authors’ telephone or E-mail address, it was difficult to contact the original authors, especially for some papers published a long time ago. So we contacted the authors of trials which were lack of randomization information and published after 2005 [9,19,22,23,42,45]. Finally, three first authors could be contacted. Only one of the authors responded that a random number table for sequence generation was used without allocation concealment, none of the participants withdrew during the trial, and no fund supported the trial [23]. Another two authors rejected to answer our questions. Based on the response, only one trial [23] was assessed to have a low risk of bias, and the others have a high or unclear risk of bias.

### Effectiveness and safety

We divided the 41 trials into two categories: CHM versus conventional medicine (17 trials) and CHM plus conventional medicine versus conventional medicine only (24 trials). Since there were different combinations of conventional medicine, we divided them into progesterone-based treatment, progesterone plus hCG-based treatment and conventional medicine with uncertain effect, and we reported the findings under the two categories with subgroups. The detailed effect estimates was presented in Table 1.

**Table 1:** Effect estimates of Chinese herbal medicines for recurrent miscarriage in 41 randomized trials

**Outcomes and comparisons**	**Effect estimate (Random effect model, 95% CI)**	**Studies**	**Participants**	**Study ID**
**Live birth rate**				
*CHM vs CM*				
CHM vs progesterone-based treatment	RR 1.31 [1.13, 1.52]*∆	4	481	Feng [38], Li [20], Yan [35], Zhu [25]
CHM vs progesterone plus hCG-based treatment	RR 1.25 [1.06, 1.47]*	1	158	Feng [45]
CHM vs CM with uncertain effect	RR 0.97 [0.41, 2.33]	3	214	Huang [41], Li [18], Wang [26]
*CHM plus CM vs CM*				
CHM plus CM vs progesterone-based treatment	RR 1.17 [1.06, 1.29]*∆	4	315	Huang [22], Li [46], Li [27], Pan [47]
CHM plus CM vshCG-based treatment	RR 1.20 [1.01, 1.43]*	1	75	He WH [19]
CHM plus CM vs progesterone plus hCG-based treatment	RR 1.08 [0.93, 1.25]	3	148	Cai [48], Tian [16], Zhao [23]
CHM plus CM vs CM with uncertain effect	RR 1.55 [1.16, 2.08]*∆	5	485	He GY [21], Shu [11], Sun [40], Xie [34], Ye [9]
**Embryonic developmental state**				
*CHM vs CM*				
CHM vs progesterone-based treatment	RR 1.43 [1.02, 1.99]*∆	3	253	Wu [32], Xu [33], Yang [36]
CHM vs CM with uncertain effect	RR 1.33 [0.99, 1.77]∆	6	515	Ban [39], Liu [24], Luo [17], Tang [12], Tian [29], Zhao [49]
*CHM plus CM vs CM*				
CHM plus CM vs progesterone-based treatment	RR 1.55 [1.23, 1.94]*∆	2	191	Yang [14], Zou [42]
CHM plus CM vs progesterone plus hCG-based treatment	RR 1.18 [1.05, 1.33]*∆	4	252	Hou [31], Li [10], Wang [30], Wang [37]
CHM plus CM vs CM with uncertain effect	RR 1.47 [1.03, 2.10]*	5	349	Fan [43], Li [44], Liu [15], Zhang [28], Zhou [13]

*Abbreviations*: *CI* confidence interval, *the effect estimate favors experimental group; ∆, result from Meta-analysis; *CHM* Chinese herbal medicine, *CM* conventional medicine, *hCG* human chorionic gonadotrophin.

### Live birth rate

21 trials reported live birth rate, of which eight trials compared CHM with conventional medicine, and 13 trials compared CHM plus conventional medicine with conventional medicine.

In trials comparing CHM with conventional medicine, four trials found a superior effect of CHM over progesterone-based treatment (RR: 1.31, 95% CI: 1.13 to 1.52; *I*^2^ = 55%, random effect model), one trial found CHM was superior to hCG-based treatment. Three trials reported CHM versus conventional medicine with uncertain effect, and two of them favored CHM. We do not pooled the data due to considerable heterogeneity (*I*^2^ = 93%).

In CHM plus conventional medicine versus conventional medicine category, four trials found a superior effect of CHM plus progesterone-based treatment over progesterone-based treatment alone (RR: 1.17, 95% CI: 1.07 to 1.29; *I*^2^ = 0%, random effect model), and one trial favored CHM plus hCG-based treatment over hCG-based treatment alone. Three trials comparing CHM plus progesterone and hCG-based treatment versus conventional medicine demonstrated no significant difference between groups (RR: 1.08, 95% CI: 0.93 to 1.25; *I*^2^ = 40%, random effect model). Five trials favored CHM plus conventional medicine with uncertain effect over conventional medicine (RR: 1.55, 95% CI: 1.16 to 2.08; *I*^2^ = 80%, random effect model).

### Embryonic developmental state

20 trials reported the embryonic developmental state in terms of effective rate, of which nine trials compared CHM versus conventional medicine and 11 trials compared CHM plus conventional medicine versus conventional medicine alone.

In trials comparing CHM with conventional medicine, three trials found a superior effect of CHM over conventional medicine (RR: 1.43, 95% CI: 1.02 to 1.99; *I*^2^ = 71%, random effect model), including a matched placebo trial comparing *Yunkang* Oral Liquid with progesterone [36]; 6 trials found comparing CHM versus conventional medicine with uncertain effect demonstrated no significant difference between two groups (RR: 1.33, 95% CI: 0.99 to 1.77; *I*^2^ = 83%, random effect model).

In trials of CHM plus conventional medicine versus conventional medicine, two trials found a superior effect of CHM plus progesterone-based treatment over conventional medicine alone (RR: 1.55, 95% CI: 1.23 to 1.94; *I*^2^ = 35%, random effect model). Four trials favored CHM plus hCG-based treatment (RR: 1.18, 95% CI: 1.05 to 1.33; *I*^2^ = 0%, random effect model). Five trials did not found significant difference between groups except one, comparing CHM plus conventional medicine with uncertain effect to conventional medicine alone. We do not pooled the data due to considerable heterogeneity (*I*^2^ = 90%).

**Figure 2:**
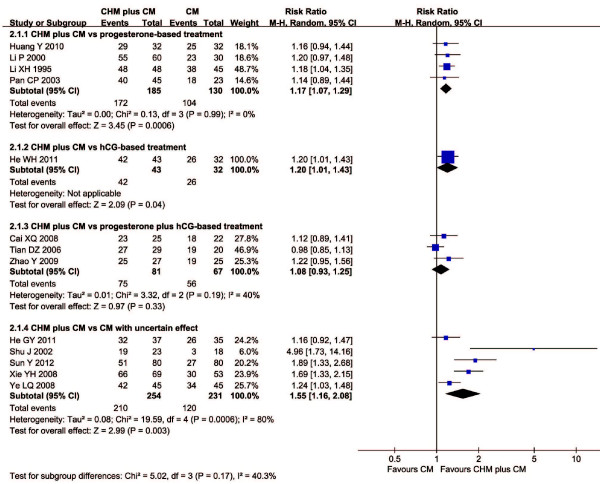
**Forest plot of CHM plus CM *versus *CM for live birth rate.** Presentation of the forest plot of Chinese herbal medicine plus conventional medicine *versus* conventional medicine for the outcome of live birth rate.

**Figure 3:**
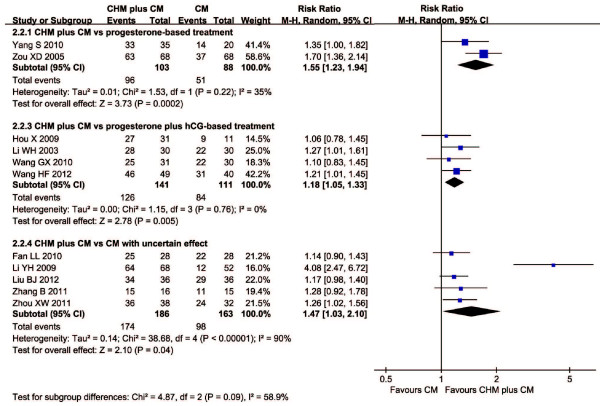
**Forest plot of CHM plus CM *versus *CM for embryonic developmental state.** Presentation of the forest plot of Chinese herbal medicine plus conventional medicine *versus* conventional medicine for the outcome of embryonic developmental state.

The forest plots of comparison of CHM plus conventional medicine versus conventional medicine alone for the outcome of live birth rate and embryonic developmental state were shown in Figures 2 and 3 respectively.

### Adverse events

Five trials reported that no adverse events occurred in CHM group [18,28,29,36,38]; six trials reported that no adverse events occurred in both CHM group and conventional group [17,20,21,23,32,44]. Two trials reported adverse events, including minor nausea in CHM group [22], and a case of fetal malformation in conventional group which used progesterone, vitamin E and some sedatives [35]. The remaining 30 trials did not report the information of adverse events.

### Funnel plot

**Figure 4:**
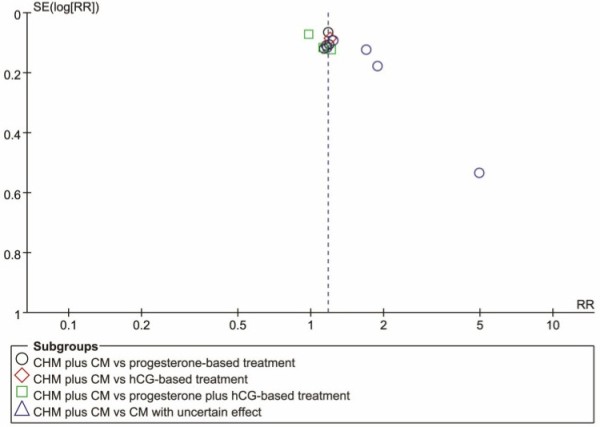
Funnel plot.

Funnel plot analysis of the thirteen studies which reported live birth rate comparing CHM plus conventional medicine with conventional medicine was performed to explore publication bias (Figure 4). The plot was asymmetrical indicating significant bias.

## Discussion

### Summary of findings

Though CHM is widely used for recurrent miscarriage in China and other eastern countries [50], there has been no evidence to support the use of CHMs for this disease.

According to this review, several CHMs, such as modified ‘*Shou Tai Wan*’ and modified ‘*An Tai Yin*’, demonstrated potentially positive effect and safety for recurrent miscarriage on improving live birth rate and embryonic developmental state. However, there was considerable clinical heterogeneity among these “positive” CHMs, due to ‘modification’ of formula. In addition, the methodological quality of these trials was generally low. Therefore, there is insufficient evidence to recommend any specific CHMs for recurrent miscarriage.

The most frequently applied formula in this review was modified ‘*Shou Tai Wan*’. In traditional Chinese medicine, the deficiency of the spleen and kidney is a major reason of recurrent miscarriage, and ‘*Shou Tai Wan*’, with the function of supplementing the kidney, fortifying the spleen, enhancing qi and nourishing blood, has been used for recurrent miscarriage in TCM since *Qing* Dynasty [51]. Accordingly, the majority of CHMs for recurrent miscarriage in this review were prescribed based on the principle of supplementing the spleen and kidney.

### Limitation of the systematic review

None of the included trials reported negative outcome and the asymmetry of the funnel plot indicated potential publication bias in this systematic review, since trials with positive results are published more easily than those with negative findings [52]. Although we tried to search the trials as systematically and comprehensively as possible, all of the trials were published in Chinese journals. Since we searched the papers in language of English and Chinese, papers published in other language, such as Korean and Japanese (CHM is commonly used in this east-Asia countries), may not be identified and included in this review, which may lead to a bias of the results.

There was a high heterogeneity in the 41 included trials: 1) baseline information was not adequately reported in some trials; 2) interventions differed from each other in treatment and control groups; comparisons were inappropriate in some trials, including using multiple interventions causing the difficulty of evaluating CHM’s specific effect and safety; 3) some controls with uncertain effect: some conventional medicines were ineffective or even harmful, such as phenobarbital [38], since studies proved that pregnancy women’ exposure to phenobarbital may causing intelligence deficits in adult [53]; 4) lack of placebo control; 5) outcomes differed in these trials: only 19 trials reported live birth rate, but other trials had a insufficient duration of follow-up and couldn’t achieve the final outcome, limiting the application of their research results.

### Implications for future research

For further research, we highlighted four issues which should be taken into consideration for Chinese medicine researches: 1) Design and reporting of RCTs on CHM for recurrent miscarriage should adhere to the CONSORT statement [54]: Sample size should be based on enough statistical power, and calculation of sample size method should be reported in the text; Randomization methods need to be described sufficiently, with an appropriate concealment; Baseline information should be reported in details; 2) Treatment intervention should be CHM decoction or Chinese patent medicine alone to be evaluated; if researchers consider a combination of CHM and conventional medicine, the conventional medicine should not be a combination of different conventional treatments, and the same conventional medicine should be used in both arms; 3) Placebo can be used for control if the intervention of treatment group is Chinese patent medicine; 4) The course of follow-up should be long enough, so as to observe and evaluate the end-point outcome (live birth or abortion) of the disease.

Last but not least, we recommend that trial protocols of CHM for recurrent miscarriage should be registered internationally and reported transparently.

## Conclusions

Some CHMs or in combination with progesterone-based treatment, may have beneficial effect on increasing live birth rate and improving embryonic developmental state for women with recurrent miscarriage. However, due to the substantial heterogeneity among herbal interventions, we should make the interpretation of the findings with caution. When comparing with progesterone and hCG-based treatment, CHM plus conventional medicine only showed superior effect in embryonic developmental state. In addition, the methodological flaws of the included trials and inconsistent findings disable us to recommend any specific CHM for recurrent miscarriage. Further clinical evidence of robust design is warranted to evaluate the efficacy and safety of CHM for the treatment of recurrent miscarriage.

## Competing interests

The authors declare that they have no competing interest.

## Authors’ contributions

GYY participated in data extraction, performed the statistical analysis, and drafted the manuscript. HL participated in search strategies development, study selection, data extraction, data analysis, and helped to draft the manuscript. XL participated in search strategies development, study selection and revised the manuscript. JPL conceived of the study, participated in its design, verified data extraction and analyses, and revised the manuscript. All authors read and approved the final manuscript.

## Pre-publication history

The pre-publication history for this paper can be accessed here:

http://www.biomedcentral.com/1472-6882/13/320/prepub

## Supplementary Material

Additional file 1: Table S1 Compositions of Chinese herbal medicines for recurrent miscarriage. Presentation of detailed compositions of Chinese herbal medicines for recurrent miscarriage in included randomized trials. Click here for file

Additional file 2: Table S2 Characteristics of included randomized trials on Chinese herbal medicine for recurrent miscarriage [9-49]. Click here for file

Additional file 3: Figure S1 Risk of bias graph. Presentation of review authors’ judgments about each risk of bias item presented as percentages across all included studies. Click here for file

Additional file 4: Figure S2 Risk of bias summary. Presentation of review authors’ judgments about each risk of bias item for each included study. Click here for file
