# Supplementary material for: Chinese herbal medicine for the treatment of recurrent miscarriage: a systematic review of randomized clinical trials
Source: BMC Complement Altern Med. 2013 Nov 18;13:320. doi: 10.1186/1472-6882-13-320 (PMC4225605; doi:10.1186/1472-6882-13-320)
Supplement: Additional file 1: Table S1 — Compositions of Chinese herbal medicines for recurrent miscarriage. Presentation of detailed compositions of Chinese herbal medicines for recurrent miscarriage in included randomized trials. [file 1472-6882-13-320-S1.doc]

**Table S1** Compositions of Chinese herbal medicines for recurrent miscarriage

| **Study ID** | **Name of CHM** | **Compositions** | **Preparation** |
| --- | --- | --- | --- |
| | He WH 2011[9] | | --- | | Modified Bushen Guchong Wan;  Practitioner-prescribed herbal formula;  Modified Bushen Antai Yin | Radix Pseudostellariae, Atractylodes Macrocephala Koidz., Rehmannia Glutinosa (Gaertn.) Libosch., Colla Corii Asini, Herba Hemiboea, Radix Scutellariae, Cortex Eucommiae, Herba Taxilli, Dipsacus Asper Wall, Rhizom Dioscoreae, Fruit of Villous Amomum, Semen Cuscutae Chinensis. | Decoction |
| Li Q 2011[10] | Modified Shou Tai Wan | Herba Taxilli, Dipsacus Asper Wall, Semen Cuscutae Chinensis, Colla Corii Asini, Radix Astragali, Radix Codonopsis Canescentis, Atractylodes Macrocephala Koidz., Poria, Radix Angelicae Sinensis, Cortex Eucommiae, Radix Scutellariae, Glycyrrhiza Uralensis Fisch. | Decoction |
| He GY 2011[11] | Modified Shou Tai Wan | Semen Cuscutae Chinensis, Herba Taxilli, Dipsacus Asper Wall, Colla Corii Asini, Radix Astragali, Boehmeria Nirea (L.) Gaud. | Decoction |
| Huang Y 2010[12] | Modified An Tai Tang | Radix Codonopsis Canescentis, Radix Astragali, Rehmannia Glutinosa (Gaertn.) Libosch., Atractylodes Macrocephala Koidz., Herba Hemiboea, Semen Cuscutae Chinensis, Dipsacus Asper Wall, Radix Scutellariae, Radix Angelicae Sinensis, Fruit of Villous Amomum | Decoction |
| | Zhao R 2009[13] | | --- | | Modified Yangxin Antai Yin | Glycyrrhiza Uralensis Fisch, Poria, Cortex et Radix Polygalae, Semen Platycladi, Ziziphus jujuba Mill, Fructus Schisandrae, Semen Cuscutae Chinensis, Herba Taxilli, Dipsacus Asper Wall, Cortex Eucommiae. | Decoction |
| Ye LQ 2008[14] | Practitioner-prescribed herbal formula | Cortex Eucommiae, Semen Cuscutae Chinensis, Dipsacus Asper Wall, Herba Taxilli, Atractylodes Macrocephala Koidz., Furctus Lycii Barbari, RadixSalviae Miltiorrhizae, Radix Angelicae Sinensis, Rhizom Dioscoreae. | Decoction |
| Cai XQ 2008[15] | Modified An Tai Yin | Semen Cuscutae Chinensis, Dipsacus Asper Wall, Herba Taxilli, Furctus Lycii Barbari, Cortex Eucommiae, Atractylodes Macrocephala Koidz., Poria, Radix Astragali, Radix Codonopsis Canescentis, Rhizom Dioscoreae, Pedicellus Cucurbitae, Boehmeria Nirea (L.) Gaud, Pericarpium Citri Tangerinae, Fruit of Villous Amomum. | Decoction |
| Xie YH 2008[16] | 1.Bushen Tiaojing Tang  2.Modified An Tai Yin | 1.Radix Codonopsis Canescentis, Radix Astragali, Atractylodes Macrocephala Koidz., Radix Angelicae Sinensis, Dipsacus Asper Wall, Rehmannia Glutinosa (Gaertn.) Libosch., Semen Cuscutae Chinensis, Fructus Rubi, Furctus Lycii Barbari, Fructus Ligustri Lucidi);  2. Radix Codonopsis Canescentis, Radix Astragali, Dipsacus Asper Wall, Semen Cuscutae Chinensis, Herba Taxilli, Atractylodes Macrocephala Koidz., Herba Hemiboea, Fructus Psoraleae, Cortex Eucommiae, Morinda officinalis How, Poria, Rhizom Dioscoreae, Glycyrrhiza Uralensis Fisch. | Decoction |
| Tian DZ 2006[17] | Zishen Yutai Pill | Radix Codonopsis Canescentis, Dipsacus Asper Wall, Atractylodes Macrocephala Koidz., Semen Cuscutae Chinensis, Herba Taxilli, Morinda officinalis How, Polygonum Multiflorum Thunb., Cortex Eucommiae, Fruit of Villous Amomum, Rehmannia Glutinosa (Gaertn.) Libosch., Furctus Lycii Barbari. | Pill, patent medicine |
| Liu HL 2005[18] | Yiqi Gushen Tang | Radix Scutellariae, Radix Bupleuri, Rehmannia Glutinosa (Gaertn.) Libosch., Rhizom Dioscoreae, Radix Astragali, Atractylodes Macrocephala Koidz., Fructus Corni, Dipsacus Asper Wall, Semen Cuscutae Chinensis, Furctus Lycii Barbari. | Decoction |
| Zou XD 2005[19] | Practitioner-prescribed herbal formula | Radix Codonopsis Canescentis, Radix Astragali, Atractylodes Macrocephala Koidz., Dipsacus Asper Wall, Radix Angelicae Sinensis, Poria, Herba Hemiboea, Rehmannia Glutinosa (Gaertn.) Libosch., Ligusticum Wallichii Franch., Glycyrrhiza Uralensis Fisch); decoction for kidney deficiency (Semen Cuscutae Chinensis, Herba Taxilli, Dipsacus Asper Wall, Colla Corii Asini, Cortex Eucommiae, Rhizoma Cibotii, Folinum Artemisiae Argyi), decoction for blood heat (Herba Hemiboea, Atractylodes Macrocephala Koidz., Rhizom Dioscoreae, Dipsacus Asper Wall, Radix Astragali, Phellodendron Amurense Rupr., Glycyrrhiza Uralensis Fisch, Radix Notoginseng), decoction for bruises (Semen Cuscutae Chinensis, Cortex Eucommiae, Os Sepiae, Ligusticum Wallichii Franch., Herba Taxilli, Radix Rubiae, Fructus Psoraleae, Fructus Ligustri Lucidi, Herba Echiptae, Trachycarpus Wagnerianus Beec. | Decoction |
| Zhu HP 2005[20] | Bushen Gutai Tang | Semen Cuscutae Chinensis, Boehmeria Nirea (L.) Gaud, Dipsacus Asper Wall, Herba Taxilli, Fructus Corni, Furctus Lycii Barbari, Atractylodes Macrocephala Koidz., Herba Hemiboea, Fruit of Villous Amomum, Radix Scutellariae, Radix Astragali. | Decoction |
| Feng H 2005[21] | 1.Chinese herbal ointment;  2.Modified Shou Tai Wan | 1. Colla Corii Asini, Folinum Artemisiae Argyi, etc.;  2. Semen Cuscutae Chinensis, Herba Taxilli, Dipsacus Asper Wall, Colla Corii Asini. | 1.Ointment;  2.Decoction |
| Pan CP 2003[22] | Modified Shou Tai Wan | Radix Codonopsis Canescentis, Radix Astragali, Semen Cuscutae Chinensis, Herba Taxilli, Dipsacus Asper Wall, Atractylodes Macrocephala Koidz., Herba Hemiboea, Glycyrrhiza Uralensis Fisch. | Decoction |
| Yan KB 2003[23] | 1.Modified Zhuyun Ertian Tang;  2. Modified Taishan Panshi San | 1. Rehmannia Glutinosa (Gaertn.) Libosch., Fructus Psoraleae, Cortex Eucommiae, Semen Cuscutae Chinensis, Radix Pseudostellariae, Radix Astragali, Atractylodes Macrocephala Koidz., Herba Taxilli, Furctus Lycii Barbari, Radix Angelicae Sinensis, Fructus Corni, Fruit of Villous Amomum, Fructus Jujubae;  2. Radix Pseudostellariae, Radix Astragali, Cortex Eucommiae, Herba Taxilli, Fructus Corni, Radix Angelicae Sinensis, Herba Hemiboea, Fruit of Villous Amomum, Colla Corii Asini, Atractylodes Macrocephala Koidz., Semen Cuscutae Chinensis, Radix Scutellariae, Furctus Lycii Barbari. | 1.Decoction;  2. Powder |
| Li WH 2003[24] | An Tai Capsule | Herba Taxilli, Dipsacus Asper Wall, Semen Cuscutae Chinensis, Radix Codonopsis Canescentis, Atractylodes Macrocephala Koidz., Herba Hemiboea, Fructus Ligustri Lucidi, Polygonum Multiflorum Thunb., Boehmeria Nirea (L.) Gaud, Pericarpium Citri Tangerinae. | Capsule, made in hospital preparation room |
| Shu J 2002[25] | Practitioner-prescribed herbal formula | Radix Codonopsis Canescentis, Radix Astragali, Radix Angelicae Sinensis, Atractylodes Macrocephala Koidz., Phellodendron Amurense Rupr., Plantula Artemisiae Capillaris, Rhizoma Anemarrhenae, Radix Scutellariae, RadixSalviae Miltiorrhizae, Poria. | Decoction |
| Li P 2000[26] | Practitioner-prescribed herbal formula | Radix Codonopsis Canescentis, Atractylodes Macrocephala Koidz., Cortex Eucommiae, Dipsacus Asper Wall, Rhizoma Cibotii, Colla Corii Asini, Folinum Artemisiae Argyi, Semen Cuscutae Chinensis, Herba Taxilli, Fructus Alpiniae Oxyphyllae, Fructus Psoraleae. | Decoction |
| Li XY 1998[27] | Try to pregnancy | Practitioner-prescribed decoction (Radix Astragali, Radix Ginseng, Radix Bupleuri, Isatis Indigotica Fort.) | Decoction |
| Feng CF 1998[28] | You Sheng Ling | Practitioner-prescribed decoction (Colla Corii Asini, Cortex Eucommiae, Semen Cuscutae Chinensis, Dipsacus Asper Wall, Herba Taxilli) | Pill, made in hospital preparation room |
| Wang YH 1997[29] | Practitioner-prescribed herbal formula | Citrus Aurantium L., Cortex Magnoliae Officinalis, Semen Cuscutae Chinensis, Colla Corii Asini, Schizonepeta Tenuifolia (Benth.) Briq., Atractylodes Macrocephala Koidz., Fruit of Villous Amomum, Bulbus Fritillariae Cirrhosae, Radix Angelicae Sinensis, Ligusticum Wallichii Franch., Rehmannia Glutinosa (Gaertn.) Libosch., Herba Hemiboea. | Decoction |
| Li XH 1995[30] | Modified Shou Tai Wan | Semen Cuscutae Chinensis, Herba Taxilli, Dipsacus Asper Wall, Colla Corii Asini. | Decoction |
| Zhang B 2011[31] | Practitioner-prescribed herbal formula | Unclear | Decoction |
| Zhou XW 2011[32] | Bushen Antai Chongji | Semen Cuscutae Chinensis, Herba Taxilli, Dipsacus Asper Wall, Radix Pseudostellariae, Atractylodes Macrocephala Koidz., Rehmannia Glutinosa (Gaertn.) Libosch., Boehmeria Nirea (L.) Gaud, Herba Echiptae. | Granules, made in hospital preparation room |
| Tian C 2011[33] | Antai Zhongzi Wan | Radix Codonopsis Canescentis, Radix Angelicae Sinensis, Herba Hemiboea, Atractylodes Macrocephala Koidz., Dipsacus Asper Wall, Cortex Eucommiae, Semen Cuscutae Chinensis, Radix Scutellariae. | Pill, made in hospital preparation room |
| Fan LL 2010[34] | Modified Shou Tai Wan | Semen Cuscutae Chinensis, Herba Taxilli, Dipsacus Asper Wall, Colla Corii Asini, Cortex Eucommiae, Furctus Lycii Barbari, Atractylodes Macrocephala Koidz., Pericarpium Citri Tangerinae. | Decoction |
| Yang S 2010[35] | Taishan Panshi San;  Modified Shou Tai Wan | Radix Ginseng, Radix Astragali, Radix Angelicae Sinensis, Dipsacus Asper Wall, Radix Scutellariae, Ligusticum Wallichii Franch., Herba Hemiboea, Rehmannia Glutinosa (Gaertn.) Libosch., Atractylodes Macrocephala Koidz., Fruit of Villous Amomum, Glycyrrhiza Uralensis Fisch, Oryza Glutinosa, Semen Cuscutae Chinensis, Herba Taxilli, Colla Corii Asini. | Decoction |
| Wang GX 2010[36] | Andian Ertian Tang | Radix Codonopsis Canescentis, Rehmannia Glutinosa (Gaertn.) Libosch., Rhizom Dioscoreae, Fructus Corni, Furctus Lycii Barbari, Fructus Ligustri Lucidi, Cortex Eucommiae, Atractylodes Macrocephala Koidz., Semen Lablab Album, Glycyrrhiza Uralensis Fisch. | Decoction |
| Hou X 2009[37] | Modified Shoutai Ertian Tang | Radix Codonopsis Canescentis, Herba Taxilli, Rehmannia Glutinosa (Gaertn.) Libosch., Atractylodes Macrocephala Koidz., Dipsacus Asper Wall, Rhizom Dioscoreae, Fructus Corni, Colla Corii Asini, Glycyrrhiza Uralensis Fisch. | Decoction |
| Luo DF 2009[38] | Practitioner-prescribed herbal formula | Ligusticum Wallichii Franch., RadixSalviae Miltiorrhizae, Radix Angelicae Sinensis, Radix Notoginseng, Pollen Typhae Angustifoliae, Faeces Trogopterori, Fructus Ligustri Lucidi, Herba Echiptae, Rehmannia Glutinosa (Gaertn.) Libosch. | Decoction, made in hospital preparation room |
| Li YH 2009[39] | Practitioner-prescribed herbal formula | Fructus Rubi, Herba Taxilli, Semen Cuscutae Chinensis, Cortex Eucommiae, Dipsacus Asper Wall, Herba Hemiboea, Radix Scutellariae, Herba Echiptae, Rhizoma Anemarrhenae, Caulis Bambusae in Taeniam, Colla Corii Asini, Radix Codonopsis Canescentis, Radix Astragali, Atractylodes Macrocephala Koidz., Herba Taxilli, Rehmannia Glutinosa (Gaertn.) Libosch., Semen Cuscutae Chinensis, Cortex Eucommiae, Dipsacus Asper Wall, Herba Hemiboea. | Decoction |
| Tang YP 2008[40] | Yikang Antai Yin | Radix Angelicae Sinensis, Atractylodes Macrocephala Koidz., Herba Hemiboea, Fructus Ligustri Lucidi, Radix Bupleuri, Cortex Moutan, Radix Scutellariae, Herba Taxilli, Boehmeria Nirea (L.) Gaud, Glycyrrhiza Uralensis Fisch | Decoction |
| Wu XY 2008[41] | Bao Tai Yin | Semen Cuscutae Chinensis, Herba Taxilli, Dipsacus Asper Wall, Polygonum Multiflorum Thunb., Radix Codonopsis Canescentis, Atractylodes Macrocephala Koidz., Rehmannia Glutinosa (Gaertn.) Libosch., Radix Astragali, Herba Hemiboea, Rhizoma Cimicifugae. | Decoction, made in hospital preparation room |
| Xu R 2007[42] | Modified Gushen Antai Tang | Semen Cuscutae Chinensis, Dipsacus Asper Wall, Cortex Eucommiae, Herba Taxilli, Radix Codonopsis Canescentis, Radix Astragali, Atractylodes Macrocephala Koidz., Fruit of Villous Amomum, Furctus Lycii Barbari, Rehmannia Glutinosa (Gaertn.) Libosch., Glycyrrhiza Uralensis Fisch. | Decoction |
| Ban YH 2003[43] | Kangyun Antai Yin | Dipsacus Asper Wall, Herba Taxilli, Semen Cuscutae Chinensis, Cortex Toonae Sinensis Radicis, Cortex Eucommiae, RadixSalviae Miltiorrhizae, Poria, Radix Pseudostellariae, Radix Astragali, Rhizoma Dioscoreae Nipponicae. | Decoction, made in hospital preparation room |
| Zhao K 2001[44] | Kunbao Heji | Herba Agrimoniae Pilosae, Semen Cuscutae Chinensis, Cortex Eucommiae, Dipsacus Asper Wall, Cortex Moutan, Radix Sangusorbae, Atractylodes Macrocephala Koidz. | Decoction |
| Yang XS 1998[45] | Yunkang Oral Liquid | Radix Codonopsis Canescentis, Radix Astragali, Rhizom Dioscoreae, Colla Corii Asini, Rhizoma Cibotii, Semen Cuscutae Chinensis, Dipsacus Asper Wall, Cortex Eucommiae, Atractylodes Macrocephala Koidz., Boehmeria Nirea (L.) Gaud, Folinum Artemisiae Argyi. | Oral liquid, patent medicine |
| Huang QZ 2012 [46] | Yu Tai Tang | Radix Dipsaci, Herba Taxilli, Cortex Eucommiae, Colla Corii Asini, Cornu Cervi Degelatinatum, Radix Codonopsis, Fructus Lycii, Folium Artemisiae Argyi, Radix Morindae Officinalis, Fructus Amomi, Radix et Rhizoma Ginseng, Rhizoma Atractylodis Macrocephalae, Semen Cuscutae, Radix Rehmanniae Praeparata. | Decoction |
| Liu BJ2012 [47] | Modified Shou Tai Wan | Semen Cuscutae, Herba Taxilli, Radix Dipsaci, Colla Corii Asini, Radix Astragali, Radix Codonopsis, Fructus Ligustri Lucidi, Herba Ecliptae, Radix Paeoniae Alba, Radix Scutellariae Hypericifoliae, Rhizoma Phragmitis, Rhizoma Dioscoreae, Rhizoma Atractylodis Macrocephalae, Rhizoma Cimicifugae. | Decoction |
| Sun Y2012 [48] | Modified Tiao Chong Tang | Semen Cuscutae, Fructus Lycii, Fructus Rubi, Herba Epimedii, Radix Dipsaci, Radix Angelicae Sinensis, Caulis Spatholobi, Radix et Rhizoma Salviae Miltiorrhizae, Radix Polygoni Multiflori, Fructus Liquidambaris, Rhizoma Cyperi, Rhizoma Dioscoreae, Herba Taraxaci, Caulis Sargentodoxae, Herba Patriniae, Fructus Citri Sarcodactylis, Pericarpium Citri Reticulatae, Folium Mori, Herba Ecliptae, Fructus Ligustri Lucidi, Cortex Moutan, Folium Eriobotryae. | Decoction |
| Wang HF2012 [49] | Le Yun Ning Granule | Radix Dipsaci, Cortex Eucommiae, Fructus Psoraleae, Radix Angelicae Sinensis, Radix Astragali, Radix Codonopsis, Rhizoma Atractylodis Macrocephalae, Rhizoma Dioscoreae, Radix Paeoniae Alba, Fructus Amomi. | Granule, patent medicine |

**Note:** CHM, Chinese herbal medicine.
