# Supplementary material for: Chinese herbal medicine for the treatment of recurrent miscarriage: a systematic review of randomized clinical trials
Source: BMC Complement Altern Med. 2013 Nov 18;13:320. doi: 10.1186/1472-6882-13-320 (PMC4225605; doi:10.1186/1472-6882-13-320)
Supplement: Additional file 2: Table S2 — Characteristics of included randomized trials on Chinese herbal medicine for recurrent miscarriage [9-49]. [file 1472-6882-13-320-S2.doc]

**Table 1** Characteristics of included randomized trials on Chinese herbal medicine for recurrent miscarriage

| Study ID | Etiology | Previous miscarriage (No.) | Mean age (range) yrs | Status in recruitment | Sample size (T/C) | Treatment regimen | Control regimen | Outcomes |
| --- | --- | --- | --- | --- | --- | --- | --- | --- |
| Ye LQ [9] | Positive ACA | ≥2 | T: 28.78; C: 28.82. | Already pregnant | 90(45/45) | 1. Practitioner-prescribed herbal formula (decoction), orally applied till ACA becoming negative; | Aspirin, orally applied till ACA becoming negative. | Live birth rate |
|  |  |  |  |  |  | 2. Control treatment. |  |  |
| Li WH [10] | Positive ACA | ≥2 | T: 27.6 ± 2.9; C: 27.3 ± 3.1. | Already pregnant | 60(30/30) | 1. An Tai Capsule (capsule), orally applied for 2 month; | 1.hCG injected and vitamin E orally applied for 2 months; | Embryonic developmental state*, negative conversion rate of ACA antibody |
|  |  |  |  |  |  | 2. Control treatment. | 2. Progesterone, injected if vaginal bleeding. |  |
| Shu J [11] | Positive ACA | ≥2 | T: 28.5 ± 3.1; C:27.3 ± 2.9. | Already pregnant | 41(23/18) | 1. Practitioner-prescribed herbal formula (decoction) before pregnancy for 2–3 months till ACA becoming negative; | Vitamin C, folic acid, and Gold Theragran (multivitamin formula with minerals) for unclear duration. |  |
|  |  |  |  |  |  | 2. Vitamin C, folic acid, and Gold Theragran (multivitamin formula with minerals) before pregnancy for 3 months till ACA becoming negative; |  |  |
|  |  |  |  |  |  | 3. Practitioner-prescribed herbal formula (decoction) after pregnancy for 7–10 days; |  |  |
|  |  |  |  |  |  | 4. Progesterone injected after pregnancy for one week; |  |  |
|  |  |  |  |  |  | 5. HCG, injected after pregnancy till 12 weeks of gestation. |  |  |
| Tang YP [12] | Positive blood group antibody or ACA | ≥2 | Unclear | Try to be pregnant | 160(80/80) | Yikang Antai Yin (decoction), orally applied for 3 months. | 1. Aspirin, orally applied for 3 months, if with positive ACA; | Embryonic developmental state*, endocrine indexes |
|  |  |  |  |  |  |  | 2. Vitamin C, injected for 10 days before pregnancy, and after pregnancy injected till 12 weeks of gestation if with positive blood group antibody. |  |
| Zhou XW [13] | Negative blocking antibodies | ≥3 | T: 28.44 ± 5.02; C: 29.30 ± 3.82. | Try to be pregnant | 80(40/40) | 1. Bushen Antia Chongji (granules), orally applied till the month of previous miscarriage; | 1. Active immunotherapy, injected once every three weeks for 4 times before pregnancy; | Embryonic developmental state*, positive conversion rate of blocking antibodies |
|  |  |  |  |  |  | 2. Control treatment. | 2. Active immunotherapy, injected once every three weeks after pregnancy till 16 weeks of gestation. |  |
| Yang S [14] | Luteal phase defect | ≥2 | T: 24–35; C: 25–39. | Already pregnant | 55(35/20) | 1. Modified Taishan Panshi San (decoction) and Modified Shou Tai Wan (decoction), orally applied for unclear duration; | Progesterone, injected for unclear duration. | Embryonic developmental state* |
|  |  |  |  |  |  | 2. Control treatment. |  |  |
| Liu BJ [15] | Luteal phase defect | ≥2 | T:28.2(23–40); C1:26.9(21–38;C2:27.5(22–40) | Already pregnant | 108(36/36/36) | 1. Modified Shou Tai Wan (decoction), orally applied till exceeding half a month of the time of previous miscarriage; | C1: Dydrogesterone, orally applied till 14 weeks of gestation; | Embryonic developmental state* |
|  |  |  |  |  |  | 2. Dydrogesterone, orally applied till 14 weeks of gestation. | C2: Progesterone, injected till 14 weeks of gestation. |  |
| Tian DZ [16] | Hyperprolactinemia | ≥2 | T: 29 ± 3; C: 28 ± 3. | Try to be pregnant | 56(25/31) | 1. Zishen Yutai Pill (Pill), orally applied from the end of menstrual period for 3 weeks; | 1. Bromocriptine, orally applied from the beginning of menstrual period for 45 days; | Live birth rate, endocrine indexes |
|  |  |  |  |  |  | 2. Zishen Yutai Pill and folic acid, orally applied after pregnancy till exceeding the time of previous miscarriage. | 2. Folic acid orally applied after pregnancy till exceeding the time of previous miscarriage; |  |
| Luo DF [17] | Pre-thrombosis | ≥2 | 29.4 ± 4.1 | Try to be pregnant | 80(40/40) | Practitioner-prescribed herbal formula (decoction), orally applied from one menstrual period before pregnancy till 12 weeks of gestation. | Fragmin (Dalteparin sodium) injected and Aspirin orally applied, till 12 weeks of gestation. | Embryonic developmental state*, endocrine indexes |
| Li XY [18] | HCMV infection | ≥3 | T: 27.71 ± 2.39; C: 26.26 ± 3.79. | Already pregnant | 160(80/80) | Practitioner-prescribed herbal formula (decoction), orally applied for 10–20 days. | Vitamin E, vitamin C and folic acid orally applied for 10–20 days. | Live birth rate, negative conversion rate of HCMV |
| He WH [19] | NR | ≥3 | T: 29.9(19–41); C: 28.5(22–37) | Try to be pregnant | 75(43/32) | 1. Modified Bushen Guchong Wan (decoction), orally applied at 2–3 months before pregnancy for 2–3 months; | 1.hCG, injected after pregnancy for 8 weeks; | Live birth rate |
|  |  |  |  |  |  | 2. Modified Bushen Guchong Wan (decoction) and Liqi Huayu Tang (decoction), orally applied during ovulatory period; | 2. Folic acid and vitamin E, orally applied after pregnancy for 3–6 months; |  |
|  |  |  |  |  |  | 3. Bushen Antai Yin (decoction), orally applied after basal body temperature rising and during pregnancy for 3 months or exceeding 15 days of the time of previous miscarriages; | 3. Progesterone, injected once every other day, if necessary. |  |
|  |  |  |  |  |  | 4. Control treatment. |  |  |
| Li Q [20] | NR | ≥2 | T: 21–35; C: 22-37 | Already pregnant | 60(30/30) | Modified Shou Tai Wan (decoction), orally applied after pregnancy for 34 weeks. | 1. Progesterone, injected after basal body temperature rising for 3–4 days, till 9–10 weeks of gestation; | Live birth rate |
|  |  |  |  |  |  |  | 2. Vitamin E, orally applied after pregnancy for 9–10 weeks. |  |
| He GY [21] | NR | ≥2 | T: 25.62 ± 3.59; C1:25.25 ± 3.80; C2:25.32 ± 3.89. | Try to be pregnant | 72(37/35) | 1. Control treatment; | Active immunotherapy, injected at 3 months before pregnancy till antibodies becoming positive. | Live birth rate, passive conversion rate of blocking antibodies |
|  |  |  |  |  |  | 2. Modified Shou Tai Wan (decoction), orally applied after pregnancy for 16 weeks. |  |  |
| Huang Y [22] | NR | ≥3 | T: 28.90 ± 2.54; C:28.65 ± 2.50 | Unclear | 64(32/32) | 1. Modified An Tai Tang (decoction), orally applied after pregnancy till 1 week exceeding the time of previous miscarriages; | 1. Acyclovir and spiramycin, orally applied before pregnancy for infection till antibodies becoming negative; | Live birth rate |
|  |  |  |  |  |  | 2. Control treatment. | 2. Progesterone, injected after pregnancy for 80 days of gestation. |  |
