# Supplementary figures and images for: Chinese herbal medicine for the treatment of recurrent miscarriage: a systematic review of randomized clinical trials
Source: BMC Complement Altern Med. 2013 Nov 18;13:320. doi: 10.1186/1472-6882-13-320 (PMC4225605; doi:10.1186/1472-6882-13-320)

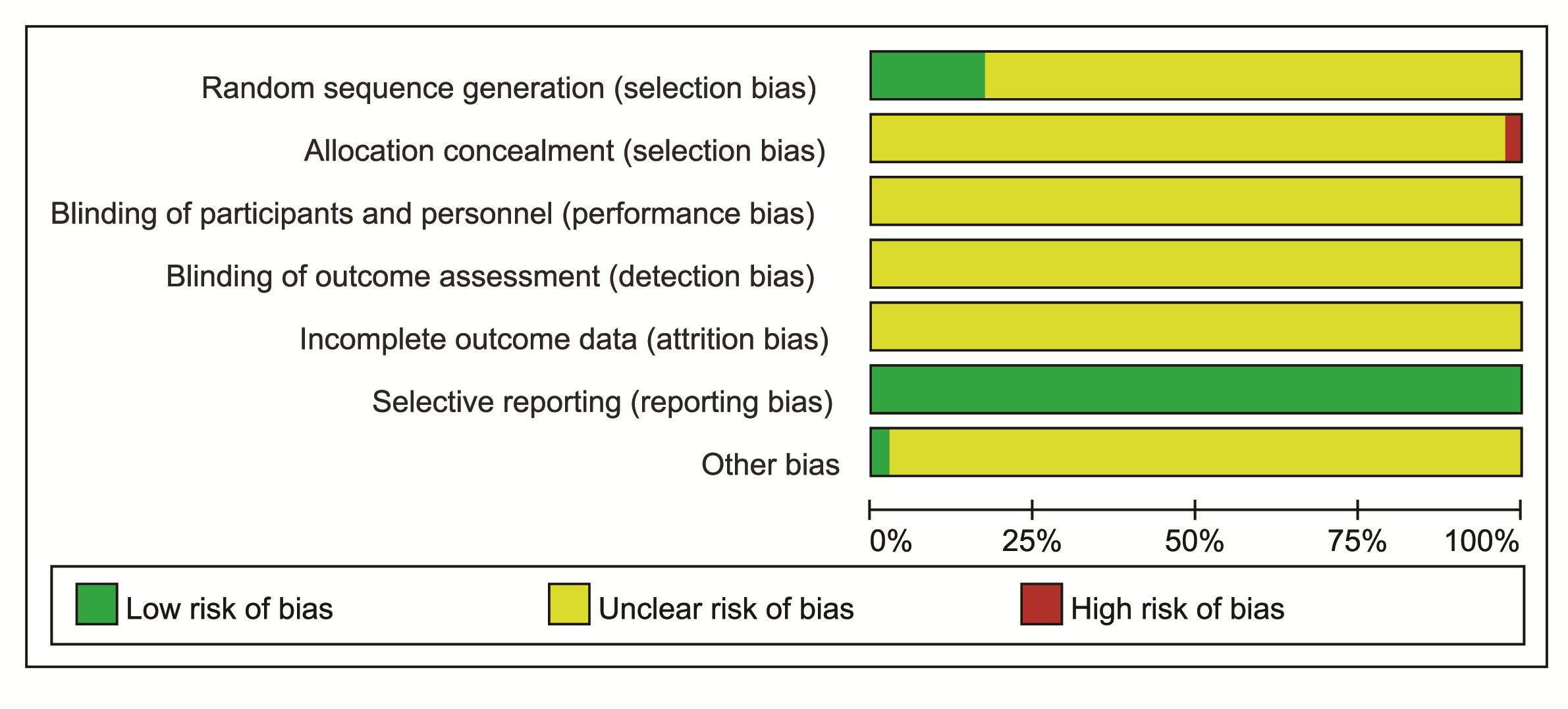

Supplement: Additional file 3: Figure S1 — Risk of bias graph. Presentation of review authors’ judgments about each risk of bias item presented as percentages across all included studies. [file 1472-6882-13-320-S3.tiff]

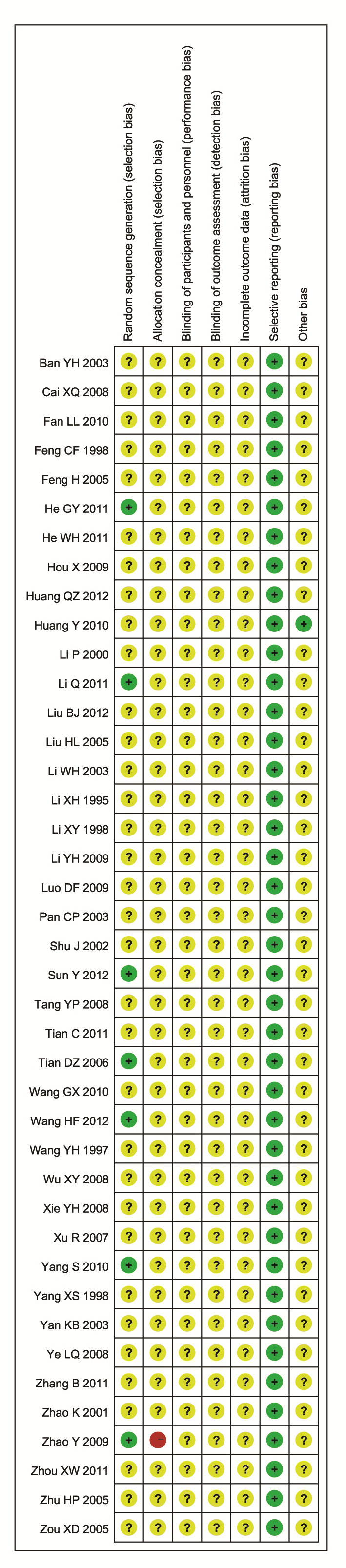

Supplement: Additional file 4: Figure S2 — Risk of bias summary. Presentation of review authors’ judgments about each risk of bias item for each included study. [file 1472-6882-13-320-S4.tiff]
